# Strain Conditions Monitoring on Corroded Prestressed Steel Strands in Beams Based on Fiber Bragg Grating Sensors

**DOI:** 10.3390/s20082288

**Published:** 2020-04-17

**Authors:** Guo-Xi Fan, Fan-Tong Lin, Peng Li, Ji-Gang Han, Huai-Shuai Shang, Ye Wang, Han Zheng

**Affiliations:** 1 School of Engineering, Ocean University of China, Qingdao 266100, China; fanguoxi@ouc.edu.cn (G.-X.F.); 21180931205@stu.ouc.edu.cn (F.-T.L.); 21180931209@stu.ouc.edu.cn (Y.W.); 17090011038@stu.ouc.edu.cn (H.Z.); 2 Liaoning Transportation Planning and Design Institute Co., Ltd., Shenyang 110111, China; hanjigang_1982@163.com; 3 School of Civil Engineering, Qingdao University of Technology, Qingdao 266033, China; shanghuaishuai@163.com

**Keywords:** FBG sensors, prestressed steel strands, corrosion, fatigue life, strain conditions

## Abstract

Fiber Bragg Grating (FBG) sensors, with excellent properties, have been widely adopted to monitor the mechanical parameters in civil engineering in recent years. On the other hand, the current study on fatigue performance of corroded prestressed steel strands is still limited, and this is mainly because the long-term strain conditions monitoring is difficult to conduct. Based on the aforementioned considerations, a total of six beam specimens were fabricated in this study. The loading mode of four points bending was adopted in the form of sinusoidal waves in the experiments. On basis of the experimental results, it can be concluded that the fatigue life of the beam decreases sharply with the increase of the corrosion rate of steel strands. Besides, with the increase of the maximum fatigue load, the fatigue life of the beam will decrease significantly. Furthermore, the existing fatigue damage of steel strand inside the beam before corrosion may further accelerate its fatigue failure. As a result, the fatigue life of the beam is reduced because of the stress concentration. Under the same external load, the strain increment and the residual strain of steel strands in the stages of loading and unloading after corrosion increase significantly compared with other stages, while the existing residual strain always shows an increasing trend at various static loading stages. Therefore, the corrosion of steel strand seriously affects not only its mechanical properties, but also its fatigue performance. Finally, the FBG sensors are capable of measuring the steel strand strain, as well as the long-term strain conditions.

## 1. Introduction

In 1997, the partially prestressed concrete (PPC) was first introduced in the European specifications. With the advantages of pretty good workability, the PPC structures have an extensive application in the fields of buildings, railway sleepers, crane beams, offshore platforms, and nuclear reactor containments in the past few years [1,2,3,4,5,6]. In addition, belonging to long-span concrete structures, ordinary bridge structures have poor ductility, heavy self-weight, and other disadvantages, while the PPC structures are ideal to overcome the disadvantages of long-span concrete structures, and this is why they have been widely applied, particularly for bridge structures [7]. During the service period of PPC bridge structures, they are not only subjected to static loads, but also inevitably exposed to the sustained and repeated loads, e.g., the vehicular loads, wave forces, wind loads, etc. Under the continuous action of repeated loads, the cumulative fatigue damage of structures will occur, which could eventually result in structural function degradation and even fatigue failure [8]. On the other hand, the fatigue damage of structures is often caused by the generated cracks under repeated loads [9]. Therefore, when referred to the PPC bridge structures with cracks, the fatigue failure events caused by repeated loads obviously attract more and more attention.

In recent years, a series of collapse accidents of prestressed concrete bridges have occurred. The investigation results show that the fatigue damage of prestressed steel bar could be accelerated by holes, moisture, and chloride ions in the prestressed duct, and then causes prestressed concrete bridge structure failure [10]. As the most widely used prestressed steel bar, the steel strand is always in the conditions of higher stress levels and smaller cross-sectional area. Thus, compared to other prestressed steel bars, the steel strand is obviously more prone to failure under the coupling effect of corrosion and fatigue load. However, previous studies focused more on the corrosion mechanism and the degradation of mechanical properties under static loads, but less on the fatigue performance of corroded steel strands. Also, few studies have been concerned with the strain conditions of corroded prestressed steel strands, which is closely related to the fatigue performance of bridge structures [11,12]. Therefore, it is necessary to study the fatigue performance of corroded prestressed steel strands and provide an effective method to monitor the strain conditions of corroded prestressed steel strands.

In the experimental studies, the commonly used resistance strain gage (RSG) has the disadvantages of poor fatigue resistance, short life, easy electromagnetic interference, apparent temperature, and zero drift circumstances, and this will lead to the premature failure of the resistance strain gage and serious distortion of measurement results during fatigue loading [13,14]. Therefore, more advanced and effective measurement methods need to be developed to monitor the strain conditions of corroded prestressed steel strands. The new type of sensor developed in the 21st century, i.e., Fiber Bragg Grating (FBG) sensor, overcomes the shortcomings of RSG sensor, and has the advantages of corrosion resistant, good stability, resistance to electromagnetic interference, high sensitivity, small size, light weight, low cost, etc., [15,16,17,18,19]. In addition, when embedded in structural members, FBG sensor has less influence on the strain conditions of corroded prestressed steel strands because of its smaller size [20,21,22]. On this basis, FBG sensor is very suitable for long-term strain conditions monitoring. By embedding a FBG sensor that has been recalibrated at cryogenic temperatures into the cement-based materials, the thermal strain of which and the factors affecting it could be effectively monitored under cryogenic temperatures for its freeze-thaw cycles [23]. Li et al. [24] proposed a novel method and relevant mechanical configurations to enhance the strain sensitivity and realize temperature compensation simultaneously for FBG-based strain sensor. The sensor developed by aforementioned authors can be used for long-term small-amplitude micro-strain monitoring in varying temperature environments. Maheshwari et al. [25] developed a novel FBG-based extensometer, which can be automated for continuous monitoring, as compared with the commonly used magnetic extensometer. In the study performed by Zheng et al. [26], the structural adhesive was used to connect the FBGs with steel wire, and the relevant measurability and reliability of the adhesive-bonded FBGs with steel wire were investigated by loading and unloading cyclic test and fatigue test in the application of FBGs to monitor the bridge cable force. The results showed that the sensors made by FBGs had good measurability and reliability. In addition, Tan et al. [27] developed a non-destructive system to monitor the rebar corrosion via FBG. For this system, FBG sensors were embedded into the specimen to monitor the expansion strain caused by rebar corrosion, and their performances were monitored by observing the Bragg wavelength shift. The test results showed that these sensors could be installed for real-time and non-destructive structural health monitoring (SHM ) monitoring in civil engineering.

As stated previously, the fatigue performance of corroded prestressed steel strands is an important challenge for bridge structures. However, the knowledge of fatigue performance of corroded prestressed steel strands is still limited, with emphasis primarily placed on static mechanical performance and less attention paid to changes in the fatigue performance of corroded prestressed steel strands. Therefore, it is necessary to study the fatigue performance of corroded prestressed steel strands and provide an effective method to monitor the strain conditions of the corroded prestressed steel strands. Based on the aforementioned considerations, a total of six beam specimens were fabricated and tested. In this study, the FBG sensors were used to monitor the strain conditions of corroded prestressed steel strands in the beam. Based on the monitoring data, the fatigue life of the beam because of the fatigue performance of corroded prestressed steel strands (steel strand broken) and the strain change rule of corroded prestressed steel strands under fatigue loads (the maximum and minimum strain increment, residual strain) were mainly analyzed, so as to provide a reference for the corrosion fatigue design of prestressed bridge structures.

## 2. Fiber Bragg Grating Sensors

### 2.1. Introduction of FBG Sensors

**Figure 1:**
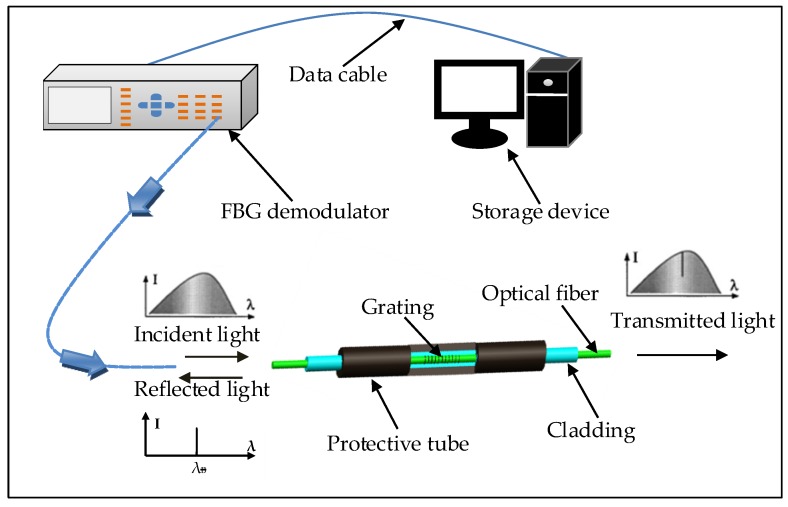
Working principle diagram of Fiber Bragg Grating (FBG) sensors.

When the broadband light from FBG demodulator is incident on the diffraction grating, only the specific wavelength corresponding to the Bragg wavelength can be reflected, and the remaining light is transmitted. Therefore, based on the changes of reflection or transmission Bragg spectrum, the absolute values of strain or temperature of the measured object can be obtained. The working principle diagram of FBG sensors is shown in Figure 1. The research of this paper is focused on the application of FBG sensors on the structure analysis, i.e., FBG sensors are used to monitor strain conditions. Thus, further research about the FBG sensors is not included in this work.

### 2.2. Relevant Parameters of FBG Sensors

The FBG sensor was written in a SMF-28e fiber by the phase-mask technique, that writes an inner core grating with a periodic refractive index. The phase-mask technique used in this experiment will put a phase-mask etched by beam exposure on an optical fiber, and the phase-mask has the function of suppressing zero order and enhancing first-order diffraction. After the phase-mask modulated, the UV laser beam is diffracted onto the fiber to form the periodic interference fringe patterns, and in the direction of paralleling to phase-mask. In addition, the wavelength of FBG sensor is recorded by the data measurement system of optical fiber sensor, which is composed of FBG demodulator, fiber fusion machine, coupler, and storage computer. The temperature data is extracted by the artificial intelligence temperature controller, and transmitted to the storage computer through the 485-usb interface. The Si425 Swept Laser Interrogator can be used to obtain the wavelength shift. Finally, the basic parameters of FBG sensors are shown in Table 1.

**Figure 2:**
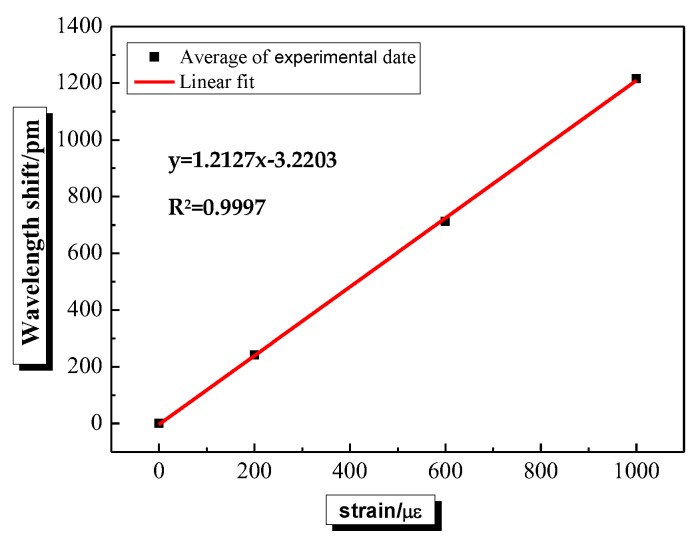
The curve of strain calibration.

**Figure 3:**
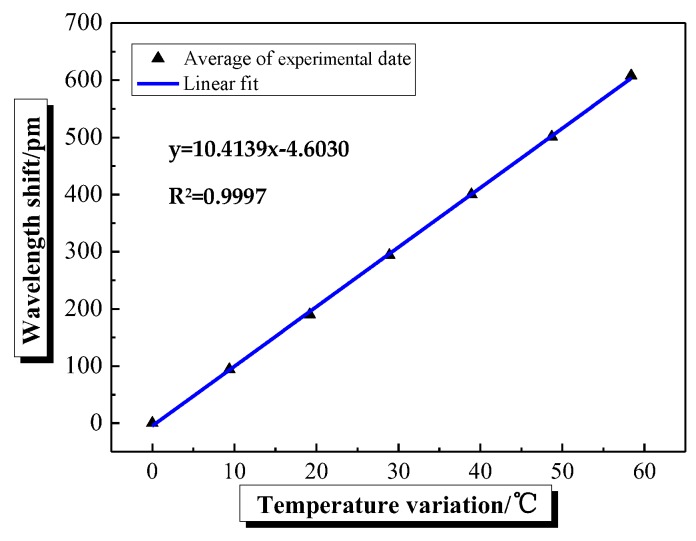
The curve of temperature response.

The strain calibration results of FBG sensors pasted into the reserved grooves on both sides of the beam are shown in Figure 2. There is a good linear relationship between the wavelength shift of FBG sensor and the strain. Besides, the correlation coefficient (R^2^) is more than 99.9%, and the strain sensitivity coefficient of FBG sensor is 1.2127 pm/με. In addition, the calibration curve of temperature response is shown in Figure 3. A good linear relationship between the wavelength shift of FBG sensor and the temperature can be also found in Figure 3. The correlation coefficient (R^2^) is more than 99.9%, and the temperature sensitivity coefficient of FBG sensor is 10.4139 pm/℃.

## 3. Experimental Program

### 3.1. Specimen Design

**Figure 4:**
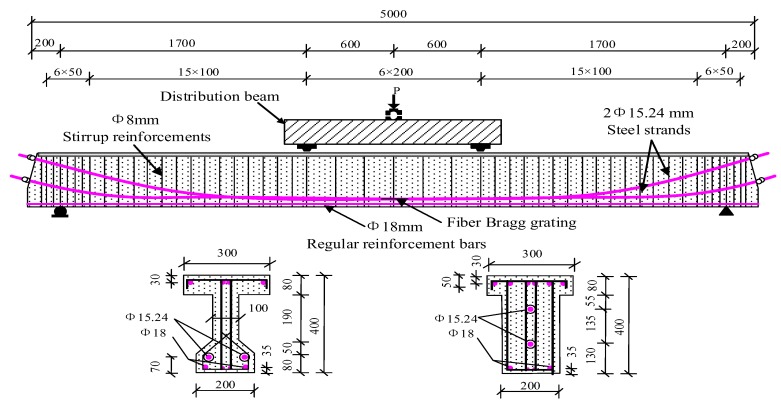
Details and loading diagram of partially prestressed concrete (PPC) beams.

A total of six specimens, i.e., post-tensioning partially prestressed concrete (PPC) beams, were fabricated with an equal amount of reinforcement bars in this experiment. The layout of reinforcement bars, cross section type, and specimen size are shown in Figure 4, respectively. In the fabrication process, the procedures of pouring concrete, tensioning steel strands, and grouting were performed in turn to prepare the desired specimens. Furthermore, the plastic corrugated pipes were used to form the reserved holes. After the age of concrete exceeding 28 days, the steel strand was tensioned along one end of the beams and fixed at the other end of the beams. Each strand with a elasticity modulus of 196 GPa was tensioned with five stages to 1306 MPa, i.e., 70% of its specified ultimate strength (i.e., 1867 MPa). After tensioning, all beams were grouted with cement mortar immediately, and the water-cement ratio was selected as 0.4 for the used cement mortar.

### 3.2. Material Properties

The same mix ratio was used for the concrete materials of the specimens, i.e., for all specimens, the weight of the water, cement, sand-coarse aggregate, and water-reducing admixture in each cubic meter of concrete material are 171 kg, 450 kg, 657.7 kg, and 270 kg, respectively. Furthermore, the water-cement ratio, percentage of sand, water reducing admixture, and design grade of used concrete material are 0.38, 0.35, 0.6%, and C50, respectively. Before conducting the specimen test, the properties of concrete material are tested. The results show that the compressive strength and tensile strength of concrete material are 60~71 MPa and 3.4~4.1 MPa, respectively. Two $7\phi_{5}^{s}$ steel strands and two HRB335 steel bars with a diameter of 18 mm are selected as the longitudinal reinforcements at the bottom of the beam. Longitudinal reinforcement bars with a diameter of 6 mm are used as erection bars, while stirrup reinforcements with a diameter of 8 mm diameter are used as shear reinforcements. Moreover, the strength grade of both erection bars and stirrup reinforcements are HPB235. The stirrup spacing is 200 mm in the loading area and 100 mm in other areas. In addition, the measured strengths of various reinforcement bars are shown in Table 2.

### 3.3. Steel Strand Corrosion

In order to facilitate the corrosion of the prestressed steel strands, a groove with a length of 20 cm was reserved in the middle of the beam span, so that the corrugated pipe will be exposed directly after beam prefabrication. Before conducting the specimen test, the corrugated pipe in the reserved groove shall be broken until the steel strand is exposed. After that, the FBG sensor will be pasted, and then the concrete material with strength grade of C30 will be backfilled. The direct current-accelerated corrosion method was adopted to accelerate the corrosion of steel strands. First, the self-made corrosion groove was pasted on the reserved backfill concrete and sealed with glass glue. Then, the sponge filled with 3.5% salt water was put into the corrosion groove. After that, the steel strands and the white steel plate were set as the anode and the cathode, respectively. Two theoretical corrosion rates (*κ*) of steel strands, i.e., 5% and 10%, were selected in this study. The time required for corrosion (*t*) can be estimated according to the Faraday’s law.

(1) $$t=\frac{2\kappa Nem_{0}}{mI}$$

where *m* is the molar mass of iron, which can be taken as 56 g/mol. *I* represents the current intensity. *m*_0_ is the mass of steel strand without corrosion. *N* is the Avogadro constant, which can be taken as 6.02 × 10^23^ mol^−1^. *e* is the elementary electric charge, which can be taken as 1.6 × 10^−19^C. The adopted current intensity (*I*) is about 0.15 A in this test, and the mass of corroded steel strand is about 220 g. According to Equation (1), it can be calculated that the time required for corrosion (*t*) is about 70 h for the theoretical corrosion rate of 5%. The time required for corrosion (*t*) is about 140 h, when the theoretical corrosion rate(*κ*) is equal to 10%.

### 3.4. Loading Program

**Figure 5:**
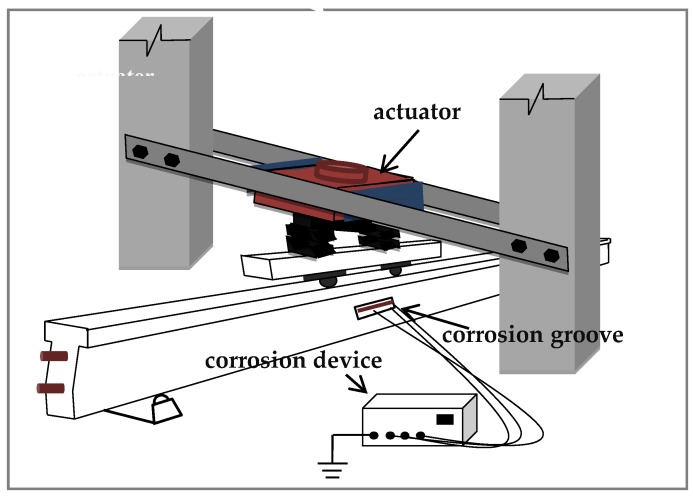
Diagram of loading and corrosion device.

A series of experiments were conducted by using the electro-hydraulic fatigue testing machine with a load capacity of 1000 kN. Loading mode of four points bending was adopted in the form of sinusoidal waves. The fatigue test loading device and corrosion device are shown in Figure 5.

The beam with identical section type and reinforcement bars was selected as the reference specimen. Based on the static loading test, the ultimate carrying capacity of the reference specimen without corrosion *F_u_* was 279 kN, which was taken as the basis of fatigue loading. The loading process is sequentially displayed as follows.

(1) The pre-cracking loading and unloading stage before corrosion. Before fatigue loading test, the static loading tests were carried out twice and applied up to the maximum fatigue load, to achieve the purpose of pre-cracking. During the static loading test, the applied load levels should be gradually increased to approximately determine the corresponding cracking load. (2) The fatigue loading stage before corrosion. Each specimen was loaded with a constant amplitude in the form of sinusoidal waves, and the loading frequency was 2 Hz. After a certain number of cycles, the loading test was stopped. Therefore, the purpose of fatigue cycle loading was gradually achieved by loading and unloading. When all the above procedures were finished, the fatigue damage of steel strands in the beam before corrosion was simulated. (3) The loading and unloading stage after corrosion. After the corrosion of the prestressed steel strand was completed, a static loading test was developed with the maximum fatigue load, so as to determine the static performance of the beam after corrosion. For each loading stage after corrosion, it should be noted that the maximum fatigue load level (*F_max_*/*F_u_*) selected for different specimens is different from each other, which can be seen from Table 3. (4) The fatigue loading stage after corrosion. The fatigue loading test under the combined action of corrosion and fatigue load was carried out until fatigue failure, and the loading frequency was 1Hz. After that, the machine was stopped and a static loading test was performed when the predetermined number of cycles was reached.

### 3.5. Installed FBG Sensors 

**Figure 6:**
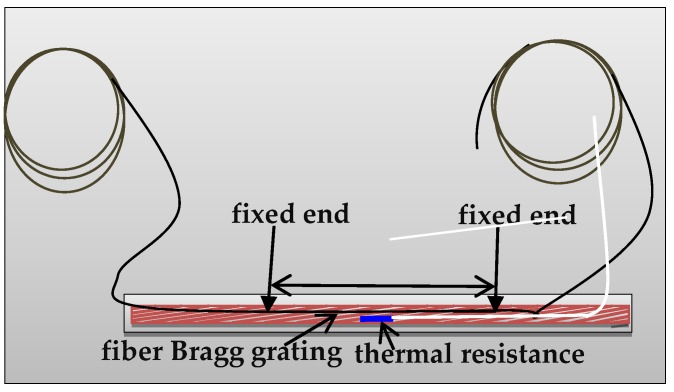
Installation position of FBG sensors.

Before the loading test, the bare optical fiber needs to be pasted on middle position of the steel strands that were located at the reserved groove on both sides of the beam, with the following procedures. First, the position for pasting the bare optical fiber was selected, as shown in Figure 4. Then, the surface of the steel strands was sanded using a sandpaper. After that, the bare optical fiber was fixed to the steel strands by using Antegu strong glue. Furthermore, in order to reduce the influence of friction on the bare optical fiber, which is induced by pouring concrete or fatigue loading, it is necessary to protect the bare optical fiber at the pasted position with epoxy resin. After curing, a small amount of butter was applied on the surface. The distance between the fixed ends, i.e., *L*, was 10 cm. Moreover, the optical fiber between the two ends is in tensioned state and parallel to the arrangement direction of the steel strand, as shown in Figure 6. To prevent the FBG sensors from failing during the accelerated corrosion process, as well as to reduce the frictional effect of concrete on the optical fiber sleeve under fatigue loading, the epoxy resin was used to protect the bare optical fiber at the adhesion point. After the epoxy resin was cured, an appropriate amount of butter was coated to the surface of the epoxy resin and the sleeve, which reduces the frictional effect and realizes the function of waterproofing. Considering the influence of temperature on the FBG sensor, the thermal resistance was embedded near the steel strand in the reserved groove and connected with the artificial intelligent temperature thermostat, so as to realize the real-time temperature monitoring. In the experiment process, the temperature change could be obtained through the above method. Then, according to the temperature sensitivity coefficient, the wavelength shift induced by the temperature change could be calculated. The wavelength shift induced by the temperature change would be removed to compensate the temperature effect of the FBG sensing results, when calculating the strain of steel strands.

## 4. Test Results and Discussion

### 4.1. Fatigue Life Analysis 

As shown in Table 3, the specimens FCF-4 and FCF-6, with a corrosion rate of 10%, failed in the loading and unloading stage after corrosion. In other words, the aforementioned specimens failed during the static loading test, while the fatigue loading test was not carried out. On the contrary, other specimens, with a corrosion rate of 5%, were not destroyed in the loading and unloading stage after corrosion. After a certain number of fatigue loading cycles, these specimens failed in the fatigue loading stage after corrosion. Therefore, the following conclusions can be drawn from Table 3. The fatigue life of the beam decreases sharply with the increase of the corrosion rate of steel strands. Besides, specimens FCF-2 and FCF-5 were loaded with an identical number of loading cycles before corrosion. Also, the maximum and the minimum fatigue loads were same with each other during this stage. However, the maximum fatigue load of the fatigue loading stage after corrosion was different from each other. The result is that the fatigue life of the beam FCF-2 is 58% lower than that of the beam FCF-5 under the combined influence of corrosion and fatigue load, which is mainly because the beam FCF-2 is under a higher fatigue load. It can be seen that the fatigue life of the beam will decrease significantly with the increase of the maximum fatigue load. Furthermore, the existing fatigue damage of steel strands inside the beam before corrosion may further accelerate its corrosion fatigue failure. The reason is that the abrasion fatigue among the steel wires could result in the surface cracking of the steel strand, and the micro-cracks gradually expand as the number of loading cycles increase. On this basis, the corrosion further increases the sizes of micro-cracks, which eventually develop into corrosion pits. Therefore, the corrosion pits will reduce the cross-sectional area of steel strands, as well as increase the actual stress. As a result, the fatigue life of the beam is reduced because of the stress concentration, e.g., specimens FCF-2 and FCF-3 were loaded with identical fatigue load levels and corrosion rates, but different number of loading cycles before corrosion of the prestressed steel strand. The loading cycles before corrosion were applied to achieve the fatigue damage of prestressed steel strand inside the beam. The result is that the fatigue life of the beam FCF-3 is reduced by 46% compared with that of the beam FCF-2, under the influence of more fatigue damage of prestressed steel strand inside the beam before corrosion.

### 4.2. Analysis of Corroded Steel Strand Strain 

The data obtained by FBG sensors in the experiment can be processed according to the following principles. It is noted that the corroded steel strand strain is the difference between the measured value and the reference value, where the latter refers to the measured wavelength before loading. The actual effective prestress of the steel strand in the beam can be estimated based on the measured concrete tensile strength and cracking moment, and then the theoretical value of steel strand strain can be obtained. Comparison of theoretical values and measured values from Table 4 shows that they match reasonably well with each other, with the ratio ranging from 0.93 to 1.05. The FBG sensors are therefore capable of measuring the steel strand strain conditions.

**Figure 7:**
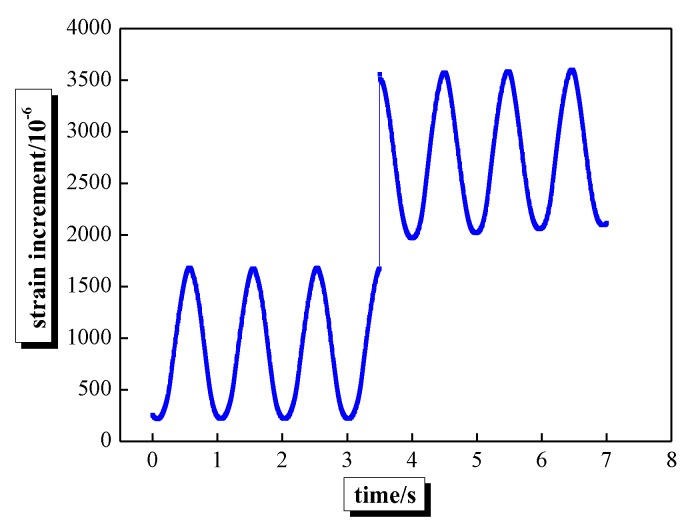
Strain increment-time curves of steel strand failure.

As shown in Figure 7, the strain increment of steel strand under fatigue load increases sharply because of the stress redistribution, when the steel strand of the beam FCF-3 is broken under fatigue load, indicating that FBG sensors can be used to monitor the corroded steel strands of the beam, also verifying its feasibility in long-term monitoring strain conditions.

**Figure 8:**
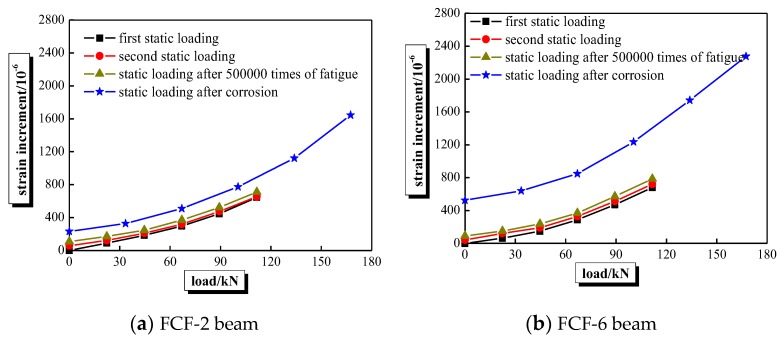
Strain-load curves of steel strands.

Figure 8 shows the steel strand strain-load curves of beams FCF-2 and FCF-6 at various static loading stages. Among which the first static loading and the second static loading correspond to the pre-cracking loading and unloading stage before corrosion, while the static loading after 500000 times of fatigue and the static loading after corrosion correspond to the fatigue loading stage before corrosion and the loading-unloading stage after corrosion, respectively. As shown in Figure 8, the bending degree of the steel strand strain-load curves is more and more obvious, indicating that the proportion of irrecoverable plastic deformation is more and more large. It can be concluded that the steel strands have residual strain at all stages, and the residual strain shows an increasing trend. Under the same external load, the strain increment and residual strain of steel strands in the stage of loading and unloading after corrosion increase significantly compared with other stages. Because of the higher theoretical corrosion rate, the strain increment and residual strain of the beam FCF-6 are significantly higher than that of the beam FCF-2, indicating that the corrosion of steel strand seriously affects its mechanical properties. Moreover, the steel strand was broken when the beam FCF-6 was loaded to the maximum fatigue load of 0.6*F_u_*, indicating that the corrosion of steel strand affects not only the mechanical properties of the beam, but also its fatigue performance.

### 4.3. Strain Variation of Steel Strand under Fatigue Load

#### 4.3.1. The Maximum and Minimum Strain Increment

**Figure 9:**
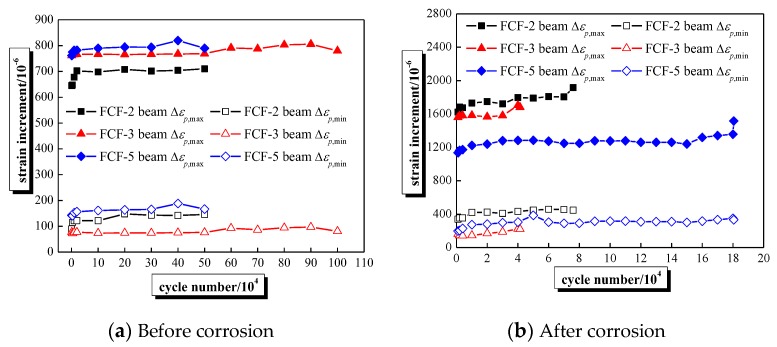
The maximum and minimum strain increment-load curves of steel strands.

The maximum and minimum strain increment of steel strand under fatigue load, i.e., Δ*ε_p,max_*, and Δ*ε_p,min_*, can be obtained by dynamic strain measured by the FBG sensors. As shown in Figure 9a, Δ*ε_p,max_*, and Δ*ε_p,min_* show similar changes for fatigue loading stage before corrosion, and they slightly increase when the load was applied 20,000 times, then, they were roughly stable as the number of loads increased. Whereas Figure 9b presents that the change of Δ*ε_p,max_* can be divided into three stages: (a) increasing stage; (b) stable stage; and (c) failure stage, e.g., the duration of the above three stages are 11%, 72%, and 17% of the fatigue life of the beam FCF-5, respectively. In addition, Δ*ε_p,min_* shows similar change of rule when fatigue load was applied before or after corrosion.

#### 4.3.2. Residual Strain

**Figure 10:**
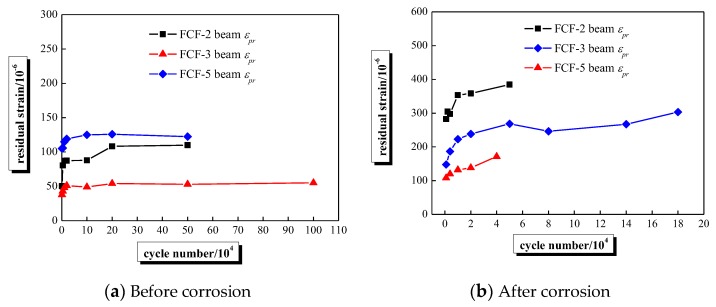
The residual strain-load cycle number curves of steel strands.

During fatigue loading, the loading process was stopped when the predetermined number of cycles was reached, while the residual strain inevitably exists in steel strands. This phenomenon is attributed to the concrete creep and the mechanical action between the steel strands and concrete aggregate at the cracking section. The corresponding change of residual strain is shown in Figure 10. The similar change of rule between the residual strain and maximum strain increment can be seen from Figure 9 and Figure 10, i.e., for fatigue loading stage before corrosion, the residual strain slightly increased when the load was applied 20,000 times, then the residual strain was not changed basically as the cycle number of fatigue loads increased. While the change of the residual strain can be divided into three stages when fatigue load was applied after corrosion, which also indicates the corrosion of steel strand can affect the fatigue performance of the beam.

According to the measured results, the maximum strain increment (Δ*ε_p,max_*), and the residual strain (*ε_pr_*) of corroded steel strands can be expressed theoretically by nonlinear regression analysis. The strain calculation equation can be expressed as follows:

(2) $$\varepsilon_{p}(n)=a{\left(n/N_{f}\right)}^{3}-b{\left(n/N_{f}\right)}^{2}+c(n/N_{f})+d$$

where, *ε_p_*(*n*) is the maximum strain increment or residual strain of the steel strand; *n* is the cyclic number of fatigue loading; *N_f_* is the number of cyclic loading when fatigue failure occurs; *a*, *b*, *c,* and *d* are constants, which are related to the number of fatigue load and the maximum load before corrosion. The related parameters of corroded steel strand mentioned in Equation (2) are listed in Table 5, where R^2^ is the correlation coefficient. The strain calculation equation can better reflect the change rules of the maximum strain increment and the residual strain of corroded steel strands under fatigue loading.

## 5. Discussion

The comparison between theoretical calculation values and experimental measurement data show that the FBG sensors are very suitable for monitoring the steel strand strain and the long-term strain conditions. On the basis of the results monitored by FBG sensors, the fatigue life and the strain conditions of the beam are studied in detail. Therefore, it can be concluded that the fatigue life of the beam is affected by the maximum fatigue load, the corrosion rate, and the fatigue damage of steel strands. Furthermore, the fatigue life of the beam will decrease significantly as the maximum fatigue load or the corrosion rate of steel strands increases. Also, both the mechanical properties and fatigue performance of the beam are seriously affected by the corrosion of steel strand. Therefore, a better agreement between the theoretical evolution rule and the above-mentioned test conclusion can be obtained by the monitored results. It is proved again that FBG sensors can be widely used in extensive fields.

## 6. Conclusions

In this study, the FBG sensors are used to monitor the strain conditions of corroded steel strands. Based on the monitored results, the conclusions could be drawn as follows.

(1) The fatigue life of the beam decreases sharply with the increase of the corrosion rate of steel strands. Besides, with the increase of the maximum fatigue load, the fatigue life of the beam will decrease significantly. Furthermore, the existing fatigue damage of steel strand inside the beam before corrosion may further accelerate its fatigue failure. As a result, the fatigue life of the beam is reduced because of the stress concentration.
(2) Comparison of the theoretical values and measured data shows that the FBG sensors are capable of measuring the steel strand strain, as well as the long-term strain conditions.
(3) At various static loading stages, the existing residual strain always shows an increasing trend. Under the same external load, the strain increment and the residual strain of steel strands in the stage of loading and unloading after corrosion increase significantly compared with other stages. The corrosion of steel strand affects not only the mechanical properties of the beam, but also its fatigue performance.
(4) The change of the maximum strain increment of steel strand under fatigue load after corrosion can be divided into three stages: (a) Increasing stage; (b) stable stage; and (c) failure stage.
(5) The maximum and minimum strain increment of steel strand show similar changes for fatigue loading stage before corrosion. The similar change of rule between the residual strain and maximum strain increment can be also found.

## Figures and Tables

**Table 1:** Parameters of FBG sensors.

Center Wavelength/nm	Reflectivity/%	3 dB Bandwidth/nm	Fiber Type	Side-mode Suppression Ratio/dB
1565.138	92.1	0.206	SMF-28e polymide	18.68

**Table 2:** Experimental results of various reinforcement bars.

Reinforcement Bars	Nominal Diameter/mm	Yield Strength *f_y_*/MPa	Ultimate Strength *f_u_*/MPa	*f_y_*/*f_u_*
steel strand	15.24	—	1867	—
HRB335	18	389	535	0.73
HPB235	8	325	480	0.68
HPB235	6	330	480	0.69

**Table 3:** Fatigue loading parameters.

Specimen		Theoretical Corrosion rate/%	*M_cr_*/kN⋅m	Before Corrosion			After Corrosion			Failure Pattern
				*F*_max_/kN	*F*_min_/kN	*N_f_*_1_/10^4^ times	*F*_max_/kN	*F*_min_/kN	*N_f_*_2_/10^4^ times	
	FCF-1	5	78	0.4 *F_u_*	0.1 *F_u_*	100	0.4 *F_u_*	0.1 *F_u_*	—	Fatigue failure, starting from steel strand broken
	FCF-2	5	84	0.4 *F_u_*	0.1 *F_u_*	50	0.6 *F_u_*	0.1 *F_u_*	7.6013	Fatigue failure, starting from steel strand broken
	FCF-3	5	72	0.4 *F_u_*	0.1 *F_u_*	100	0.6 *F_u_*	0.1 *F_u_*	4.1406	Fatigue failure, starting from steel strand broken
	FCF-4	10	77	0.4 *F_u_*	0.1 *F_u_*	100	0.4 *F_u_*	0.1 *F_u_*	—	Failure during static loading, with steel strand broken
	FCF-5	5	82	0.4 *F_u_*	0.1 *F_u_*	50	0.5 *F_u_*	0.1 *F_u_*	18.1048	Fatigue failure, starting from steel strand broken
	FCF-6	10	78	0.4 *F_u_*	0.1 *F_u_*	50	0.6 *F_u_*	0.1 *F_u_*	—	Failure during static loading, with steel strand broken

Notes: *M_cr_* is the measured cracking moment. *F*_max_ is the maximum fatigue load. *F*_min_ is the minimum fatigue load. *N_f_*_1_ and*N_f_*_2_ are the number of loading cycles before and after corrosion of the prestressed steel strand, respectively. In this paper, the cycle number which is corresponding to the sudden change in the strain of the steel strand monitored by the FBG sensor, is selected as the fatigue life of the specimen. However, the fatigue life of specimen FCF-1 cannot be given due to the failure of the FBG monitoring equipment.

**Table 4:** Comparison of calculated values and measured values of strain increment.

Beam	Calculated Value/10^−6^	Measured Value/×10^−6^	Calculated Value /Measured Value	Beam	Calculated Value/10^−6^	Measured Value/×10^−6^	Calculated Value/Measured Value
FCF-1	613	—	—	FCF-4	699	668	1.05
FCF-2	621	654	0.94	FCF-5	657	704	0.93
FCF-3	646	707	0.91	FCF-6	654	689	0.95

**Table 5:** Parameters of strain calculation equation.

Beam	*ε_p_*	*a*	*b*	*c*	*d*	*R* ^2^
FCF-2	Δ*ε_p_*_,max_	857.82	1293.7	684.87	1642.6	0.8946
	*ε_pr_*	748.54	1225	588.7	272.54	0.957
FCF-3	Δ*ε_p_*_,max_	649.05	722.12	210.33	1563.3	0.9448
	*ε_pr_*	240.16	361.11	188.21	104.07	0.9998
FCF-5	Δ*ε_p_*_,max_	1785.5	2582.7	1074.1	1149.2	0.8383
	*ε_pr_*	858.83	1384.5	663.9	169.8	0.8914
